# Pituitary adenylate cyclase-activating polypeptide plays a role in neuropsychiatric and substance use disorders: sex-specific perspective

**DOI:** 10.3389/fnins.2025.1545810

**Published:** 2025-02-05

**Authors:** Catherine E. Van Doorn, Mikala M. Zelows, Anel A. Jaramillo

**Affiliations:** Department of Pharmaceutical Sciences, College of Pharmacy, University of Kentucky, Lexington, KY, United States

**Keywords:** neuropsychiatric disorders, PACAP, drug abuse and addiction, sex differences, substance use cycle

## Abstract

The neuropeptide pituitary adenylate cyclase-activating peptide (PACAP) plays a pivotal role in regulating stress, fear, and anxiety responses. Genetic and molecular studies investigating PACAP demonstrate sex-dimorphic characteristics, with females exhibiting increased reactivity of PACAP signaling in neuropsychiatric disorders. Studies expand the role of PACAP to substance use disorders (SUD) by demonstrating modulation of PACAP can lead to neurobiological changes induced by nicotine, ethanol, stimulants and opioids. Given that females with SUD exhibit distinct drug use, relapse, and withdrawal sensitivity relative to males, we hypothesize that the PACAP system contributes to these sex-specific differences. Therefore, we review the role of PACAP in SUD by characterizing the role of PACAP at the molecular, brain regional, and behavioral levels relevant to the addiction cycle. We present literature linking PACAP to neuropsychiatric disorders, which demonstrate the intricate role of PACAP within neuronal signaling and pathways modulating addiction. We hypothesize that females are more particularly susceptible to PACAP-related changes during the intoxication and withdrawal phases of the addiction cycle. Altogether understanding the sex-specific differences in the PACAP system offers a foundation for future studies aimed at developing tailored interventions for addressing SUD.

## Introduction

Pituitary Adenylate Cyclase-Activating Polypeptide (PACAP) is a neuropeptide crucial to development, metabolism, neuroendocrine, and immune regulation. PACAP intricately modulates the hypothalamic–pituitary–adrenal (HPA) axis, which is pivotal in controlling the body’s response to stress. Notably, both PACAP peptide and its receptor, PACAP 1 receptor (PAC1R), exhibit elevated expression in brain regions associated with stress modulation responses and anxiety-related behaviors, as well as in regions involved in substance use disorder (SUD), including the hypothalamus, thalamus, bed nucleus of the stria terminalis (BNST), nucleus accumbens, parabrachial nucleus (PBN), striatum, and hippocampus (Vaudry et al., 2009; Rajbhandari et al., 2023; Boucher et al., 2021; Jaramillo et al., 2021; Lutfy and Shankar, 2019; Curtis et al., 2023). Recent studies highlight sex-specific differences in PACAP function and expression in neuropsychiatric disorders. Similarly, in SUD, PACAP signaling suggest sex-specific alterations at stages of addiction, influencing susceptibility and progression. This review builds upon current literature on SUD (Gargiulo et al., 2020; Stojakovic et al., 2020; Miles et al., 2019) and sex differences reviews (Rajbhandari et al., 2023; Bangasser and Valentino, 2014), demonstrating the sex-dimorphic role of PACAP signaling.

## PACAP characterization

### Molecular

PACAP is comprised of 27 or 38 amino acids resulting in two isoforms PACAP27 and the more abundant PACAP38 (Bangasser and Valentino, 2014). PACAP38 is approximately 10–100 times more abundant than PACAP27 in the brain, and the half-life of PACAP38 is around 5 min compared to the 45 min of PACAP27 (Vaudry et al., 2009; Miles et al., 2019; Hirabayashi et al., 2018). Both PACAP27 and PACAP38 signal using three main G-protein-coupled receptors: vasoactive intestinal peptide receptors 1 and 2 (VPAC1-R, VPAC2-R) and PAC1R (Hirabayashi et al., 2018). While PACAP27 and PACAP38 bind to the VPAC1-R and VPAC2-R receptors, both isoforms display a 1,000-fold higher affinity for the PAC1 receptor than vasoactive intestinal peptide (VIP) (Vaudry et al., 2009; Jansen-Olesen and Hougaard, 2018). VIP predominantly binds to VPAC1-R and VPAC2-R; however, this review will focus on PACAP and PAC1R due to their strong association with neuropsychiatric disorders and SUD (Vaudry et al., 2009; Miles et al., 2019; Hirabayashi et al., 2018; Jansen-Olesen and Hougaard, 2018; Ressler et al., 2011; Kormos and Gaszner, 2013; Hammack et al., 2010). Subsequent downstream signaling through PAC1R predominantly activates adenylate cyclase and cAMP signaling; however, coupling to class B1 G-protein-coupled receptors (GPCRs) G-protein subunits (e.g., G_s_-protein for adenylate cyclase; G_q/11_ and G_i/o_ for phospholipase C and intracellular calcium mobilization) results in signaling diversity (Lu et al., 2022). Additional signaling involves the recruitment of inositol triphosphate (IP3), mitogen-activated protein kinase (MAPK), and protein kinase C pathways (Vaudry et al., 2009; Hirabayashi et al., 2018; Winters and Moore, 2020). The diversity of pathways allows for multiple functional outcomes via PACAP signaling, (Yang et al., 2020) some of which are affected by SUD. Additionally, multiple variants of the PAC1R receptor exist via alternative splicing of 2 exons that respond to PACAP27 and PACAP38 binding (Winters and Moore, 2020; Yang et al., 2020; Blechman and Levkowitz, 2013; Spengler et al., 1993). There are two main PAC1R isoforms identified in humans (Vaudry et al., 2009), although isoform presence differs in a species-dependent manner (Blechman and Levkowitz, 2013; Spengler et al., 1993), with expression patterns varying across regions of the CNS and PNS (Vaudry et al., 2009). Evidence suggests sex differences in receptor isoform levels may impact PACAP signaling in the CNS and peripheral regions (Zhou et al., 2000; Barberi et al., 2007; Daniel et al., 2001). However, further studies are needed to investigate if they have different roles in neuropsychiatric disorders such as SUD.

### Neuroendocrinological differences

Data present differences in developmental and regional expression of PAC1R and PACAP, possibly associating to sex differences in SUD. Baseline sex differences are evident during development, with females exhibited higher hippocampal PAC1R mRNA levels during development and young adulthood (Shneider et al., 2010). At this stage in development, there was no difference in individual PAC1R isoform variant expression by sex; however, there could be differences in other ages, isoforms, and species (Shneider et al., 2010). Additionally, PACAP exposure occurs before birth, as PACAP is present in the amniotic human samples (Toth et al., 2020). These data suggest the critical role of PACAP signaling in development, indicating that disruptions in PACAP may lead to developmental effects. For example, pups lacking the PACAP gene *Adcyap1* (PACAP knockouts, PACAP−/−) exhibit a high mortality rate, highlighting the developmental significance of PACAP (Hashimoto et al., 2001). Below, we discuss the role of PACAP in utero and in postnatal exposure to drugs of abuse and its implications in drug phenotypes.

In adulthood, studies have identified sex differences in PACAP mRNA in the adrenal gland (higher in males) and superior cervical ganglia (higher in females), while no differences have been observed in overall hypothalamic expression (Mosca et al., 2015). This is unusual, as sub-regions of the hypothalamus (e.g., the PACAP-rich paraventricular nucleus of the hypothalamus [PVN]) could illustrate sex differences. However, the authors note that testing the whole hypothalamus could have obfuscated any specific-nuclei sex differences (Mosca et al., 2015). When comparing the paraventricular nucleus of the thalamus (PVT), females exhibit higher levels of PACAP mRNA compared to male mice (Curtis et al., 2023). Interestingly, evidence of subregion-specific expression of PACAP was found in the anterior and posterior aspects of the PVT, wherein the anterior PVT showed less PACAP mRNA than the posterior PVT for both sexes. Additional experiments found females had significantly more PACAP27+ immunofluorescent cells than males in the anterior/middle PVT and more PACAP38+ cells in both the anterior and posterior PVT (Curtis et al., 2023). Given that thalamic regions are associated with affect and motivation, differences in PACAP expression may contribute to sex differences seen in the SUD cycle (Curtis et al., 2023; Miles et al., 2019).

PAC1R and PACAP are expressed in both the central nervous system (CNS) and peripheral tissues, particularly the gonads, where significant sex differences in reproductive functions are observed. In male rats, PAC1R mRNA is present in the seminiferous tubules, with the testes exhibiting the highest concentration of PACAP38 across all body tissues (Daniel et al., 2001; Arimura et al., 1991). Additionally, the PAC1R splice variant, PAC1R(3a), expressed by Sertoli cells of the testes, demonstrates a higher affinity for PACAP38 compared to the wild-type PAC1 receptor (Daniel et al., 2001).

In humans, similar patterns are observed with high peripheral expression of PACAP in the testis, suggesting that sex differences observed in rodent models are likely applicable to humans (Nakamura et al., 2014). Additionally, PACAP has been found to impact sperm motility, testosterone production, and sexual differentiation in male mice [see (Winters and Moore, 2020) for a comprehensive review]. In female mice, PAC1R has been found in granulosa cells and ovarian tissue (Barberi et al., 2007), thereby impacting follicle growth, ovulation, and the secretion of estradiol and progesterone (Vaccari et al., 2006).

There is crosstalk in the PACAP system between the CNS and periphery, as it relates to neuronal factors/hormones. For example, when PACAP is administered via intracerebroventricular (ICV) injection, gonadotropin-releasing hormone (GnRH) increased in male rats by 12.5% that was abolished by administering PACAP antagonist PACAP(6-38) (Li et al., 1996). In female rats, PACAP mRNA expression in the PVN and pituitary was found to fluctuate with the rat estrus cycle (Moore et al., 2005). PACAP peptide was increased in ‘preovulatory follicles’ after human chorionic gonadotrophin (hCG) stimulation (Barberi et al., 2007). PACAP administered directly to the CNS via ICV injection blocks ovulation (Briscione et al., 2017; Köves et al., 2014). These studies clearly show crosstalk between PACAP in the CNS and gonadal hormones. Another example of this relationship is exemplified by estrogen’s effects on PACAP in the BNST. The BNST, a region dense in PACAP projections and PAC1R, demonstrates sex-specific characteristics in part associated with estrogen modulation. For example, OVX female rats given estrogen replacement pellet implants showed increased mRNA for PACAP (*Adcyap1*) in the BNST 2–3 fold vs. control implants (Ressler et al., 2011). It is unknown whether estrogen has the same effect in males in the BNST, although sex hormone responsiveness is seen in other regions. For example, treatment of male anterior pituitary cultured cells with progesterone or testosterone paired with GnRH increased PACAP mRNA while estradiol blocked it (Zheng et al., 2014). Additionally, there is an estrogen response element in the gene for the PAC1R, indicating a strong interaction point between PACAP signaling and gonadal hormones (Ressler et al., 2011). Given the close interaction, previous reviews hypothesized that PACAP is sensitive to glucocorticoid and estrogen in stress-inducing situations, possibly extending to SUD (King et al., 2017). These data demonstrate evidence that PACAP regulates sex hormones and has a sex-specific role in reproductive function, thereby laying the groundwork for sex differences for other physiologic functions (Koppan et al., 2022). Together, these CNS regions and peripheral areas, with their classic roles in stress and anxiety, also influence the development and perpetuation of the cycle of SUD, and each stage is likely mediated by PACAP signaling in a sex-dependent manner.

## PACAP dysregulation

### Neuropsychiatric disorders

#### Stress

Both sexes experience stress throughout their lifespan. However, their responses to stress are multifactorial. Environmental influences, genetics, and neurobiological differences lead to differences in the stress response that contribute to the development of stress-related disorders (Altemus et al., 2014). The role of the PACAP system has been extensively studied in stress (Rajbhandari et al., 2023; Boucher et al., 2021; Lutfy and Shankar, 2019), which is highly relevant to SUD. PACAP regulates the HPA axis response through corticotropin-releasing hormone (CRH) at the PVN, catecholamines from the adrenals (Eiden et al., 2018), and the release of vital stress hormones such as adrenocorticotropic hormone (ACTH) (Eiden et al., 2018; Eiden, 2013). Therefore, PACAP is vital to the neuroendocrine stress response (Rajbhandari et al., 2023; Mustafa et al., 2014). There are sex differences in HPA axis function that lead to differential responses to stress (Bangasser and Valentino, 2014). There is a gap in the literature regarding how PACAP in females specifically responds to stress. However, there are a few notable studies. In preclinical models, females exhibit higher levels of the stress hormone corticosterone at baseline and 2 h post-acute restraint stress (Figueiredo et al., 2002). PACAP38 infusion into the BNST in both sexes of rats subsequently showed increased plasma corticosterone, and while the sexes were not directly compared, females appeared to have a higher corticosterone release (Lezak et al., 2014). Males exposed to chronic variate stress showed a PACAP-potentiated corticosterone release vs. vehicle and non-stressed controls, but this was not tested in females (King et al., 2017). PACAP may mediate a greater HPA axis response in females, although the acute and chronic effects are likely different.

Changes in PACAP expression occur in response to stress. Utilizing water deprivation to elicit stress reduced PACAP38 levels in the hypothalamus of both sexes, with females showing a more drastic decrease (Kiss et al., 2007). Responses differ depending on the region studied, as whole brainstem PACAP38 increased after water deprivation in males but not females (Kiss et al., 2007). The role of PACAP on stress is also age-dependent, as neonatal-maternal separation stress did not change PACAP or PAC1R gene expression in the hypothalamus, carotid body, or superior cervical ganglia of male and female pups (Mosca et al., 2015). These studies demonstrate that stress-induced changes in PACAP levels are age-dependent and region-specific (Mosca et al., 2015; Hammack et al., 2009; Pêgo et al., 2008). As PAC1R mRNA expression is different in females at baseline in several stress-responsive tissues and regions (e.g., adrenal glands, superior cervical ganglia, PVT, hippocampus), further regulation of PACAP by cycling estrogen could help explain some of the sex differences in behavioral output (Curtis et al., 2023; Shneider et al., 2010; Mosca et al., 2015).

Repeated and chronic stress models show the importance of PACAP in the male stress response but provide limited information for females [see (Hammack and May, 2015) for review]. Chronic exposure to stress for 7 days led to an increase in both PACAP and PAC1R mRNA in the BNST of male rats (Hammack et al., 2009; Lezak et al., 2014) and resulted in increased regional volume (Hammack et al., 2009). PACAP38 ICV administration in the BNST also increases corticosterone release in both female and male rats (Lezak et al., 2014; Agarwal et al., 2005). Conversely, chronic social defeat stress in male rats elicited a 23.4% increase in PACAP densitometry and increased PAC1R mRNA in the central amygdala (CeA) but not in the BNST (Seiglie et al., 2022). However, a single social defeat bout increased PACAP densitometry in both the CeA and BNST (Seiglie et al., 2022). This is interesting as it shows the PACAP response differs by region with repeated stress exposure. Male PACAP−/− models reinforce the role of PACAP in the stress response, as PACAP−/− mice show less corticosterone, more hyperactivity, anxiolytic, and anti-depressive-like behaviors after repeated stress relative to wild-type mice (Lehmann et al., 2013). PACAP’s role in stress exhibits specificity to psychological stress, as an LPS injection in PACAP−/− mice did not result in a blunted plasma corticosterone response (Hattori et al., 2012). Given the strong association between psychological stress and SUD (Cleck and Blendy, 2008), the role of PACAP may be relevant to the perpetuation of SUD.

#### PTSD

While the symptom presentation of post-traumatic stress disorder (PTSD) does not differ between females and males, the lifetime prevalence rates are approximately 5% for males and 10% for females, indicating sex-based differences in response to trauma (Altemus et al., 2014; Lehavot et al., 2018).

Traumatic experiences in childhood and adulthood can elicit PTSD and thus onset occurs post-acute or chronic trauma, not generalizable to a specific age (Altemus et al., 2014). PACAP and polymorphisms in the PAC1R are linked to PTSD (Ressler et al., 2011). Females have greater exposure to certain types of trauma (Altemus et al., 2014) are at greater risk of developing PTSD (Briscione et al., 2017) and exhibit PACAP-specific changes compared to males. Notably, only females with PTSD exhibit elevated blood levels of PACAP38, which correlate with both the diagnosis and the number of symptoms (Ressler et al., 2011). Genetic studies further support the role of PACAP in PTSD, particularly in females. For instance, the single nucleotide polymorphism (SNP) rs2267735 in the PAC1 receptor gene (*ADCYAP1R1*) is significantly associated with PTSD diagnosis in females but not in males (Ressler et al., 2011). This SNP has also been linked to childhood maltreatment (Uddin et al., 2013) and PTSD in females (Almli et al., 2013), underscoring a gender-specific genetic vulnerability. Additionally, cortisol levels, which vary between sexes, show that only females with PTSD exhibit lower cortisol levels compared to healthy, sex-matched controls [for review, see (Bangasser and Valentino, 2014)].

Given that PACAP can modulate cortisol levels, it suggests a potential underlying mechanism for the observed sex differences in cortisol levels in females with PTSD. Higher circulating PACAP levels were associated with increased functional connectivity in the CeA in trauma-exposed adult females but not trauma-exposed adult males (Clancy et al., 2023). The increase in PACAP alongside the increased functional connectivity is thought to show how the PACAP system in the amygdala is involved in ‘threat-orienting behavior’ and may be unique to females with PTSD (Clancy et al., 2023). Additionally, there is evidence that active ovarian hormones/neurosteroids, such as estradiol, play mediatory roles in these sex disparities in females [for an in-depth review, see (Briscione et al., 2017)]. Estradiol replacement therapy or fear conditioning in OVX female mice increased PAC1R gene expression in the BNST and amygdala, respectively, demonstrating the link between PACAP, estradiol, and an abnormal stress response (Ressler et al., 2011). The same group looking at PTSD risk in females demonstrated the functional impact of the risk allele, where there was less efficient binding of the estradiol/estrogen receptor alpha complex to the estrogen response element containing the risk allele of rs2267735 *in vitro* (Mercer et al., 2016). Decreased estradiol/receptor complex binding to the estrogen response element is thought to reduce the expression of *ADCYAP1R1* (Mercer et al., 2016). As a result, there would be subsequently decreased receptor expression and reduced estrogen regulation in adaptation to stress, which in turn is thought to increase the risk for PTSD (Mercer et al., 2016). Additionally, the study found that females who have both low serum estradiol and the risk allele also had lower PAC1R gene expression and greater PTSD symptoms. Together, these findings demonstrate that the risk allele rs2267735 lowers the ability of estradiol/estrogen to modulate the PACAP system, suggesting a mechanism for sex differences in PTSD symptom severity and diagnosis (Mercer et al., 2016). Considering the increased risk for females to develop PTSD and the SNP within the estrogen response element in the *ADCYAP1R1* gene, this demonstrates an additional point of vulnerability for females, and PACAP strongly appears to be a part of this risk in PTSD. Given the role of PTSD in susceptibility to SUD, the role of PACAP in PTSD is highly relevant to SUD.

#### Anxiety

Anxiety disorders are twice as prevalent in females as in males (Altemus et al., 2014). Notably, sex differences in the initial presentation of anxiety manifest early, beginning prepubertally in childhood. However, the onset of generalized anxiety disorder (GAD) typically occurs in the third decade of life for both females and males (Altemus et al., 2014). Females with GAD often experience symptoms such as fatigue, muscle tension, cardiorespiratory issues, and gastrointestinal symptoms. In contrast, males are more likely to suffer from a negative impact on relationships and are more prone to concurrent SUD (Altemus et al., 2014). The role of PACAP in anxiety and its influence on these sex-specific manifestations have been extensively reviewed, underscoring its significant impact on the pathophysiology of anxiety disorders (Boucher et al., 2021; Lutfy and Shankar, 2019). Briefly, data in males consistently show that PACAP is pro-anxiety-like and that genetic PACAP−/− knockout animals are protected against these effects. PACAP and PAC1R gene expression are increased alongside anxiety-like behaviors (King et al., 2017; Hammack et al., 2009; Lehmann et al., 2013; Dore et al., 2013). In male rats, chronic social defeat increased anxiety-like behavior in the light–dark test, which was reversed in CeA viral-mediated knockdown (Seiglie et al., 2022). Chronic variable mild stress-induced anxiety-like behaviors in EPM, open field, and NSFT (Hu et al., 2020). Moreover, activation of the PACAP system can directly induce anxiety-like behavior. In males, ICV PACAP38 increased anxiety-like behaviors in the elevated plus maze (EPM) (Dore et al., 2013). Specific infusion of PACAP into the CeA or BNST also produced similar results in EPM (Iemolo et al., 2016; Roman et al., 2014). Alternatively, in the habenula, chemogenetic activation of PACAP+ neurons decreased anxiety-like behaviors in an open field in both sexes of mice (Levinstein et al., 2022). Male PACAP−/− mice showed anxiolytic responses in open field, EPM, emergence, and novel-object tests (Hashimoto et al., 2001; Hattori et al., 2012). Social defeat stress-exposed PACAP−/− mice showed anxiolytic responses in light–dark and elevated zero maze compared to defeated wild-type mice (Lehmann et al., 2013). Additionally, male PACAP−/− mice show anxiolytic responses in a novel environment, increased social interaction, and lower plasma corticosterone after restraint or chronic social defeat stress exposure (Lehmann et al., 2013; Hattori et al., 2012). PACAP−/− (sex unspecified) show notable psychomotor behaviors such as ‘explosive jumping behaviors,’ hyperlocomotion/hyperactivity, and exploratory behavior in a non-home cage, open field environment (Hashimoto et al., 2001). The disrupted locomotor and anxiety-like behaviors were reversed with antipsychotic haloperidol (Hashimoto et al., 2001). This phenotype may be mediated through the locus coeruleus, as the selective deletion of PAC1R in this region led to the anxiolytic and hyperactive phenotypes observed in an open field and EPM tests, similar to other knockout models in males (Nguyen et al., 2024). The influence of PACAP on psychomotor effects is also shown in SUD and is discussed later in this review. Less is known about PACAP and anxiety in females, however there are some interesting sex differences that show the role of PACAP in anxiety may also be sex-specific. For example, in naturally cycling female rats, chronic variable stress followed by a PACAP infusion into the BNST resulted in a reduced acoustic startle response compared to baseline, as opposed to males, who showed a potentiated startle response (King et al., 2017). Notably, PACAP infusion before testing showed an enhanced startle response in non-stressed female controls. Chronic stress exposure likely modified the PACAP system response differently between the sexes, resulting in a difference in the anxiety-related behavioral outcome (King et al., 2017).

In humans with generalized anxiety disorder (GAD), circulating PACAP38 levels were significantly lower in females with GAD compared to healthy female controls (Ross et al., 2020). In direct contrast, there was no difference in circulating PACAP38 in males with GAD vs. male healthy controls. Interestingly, the same PAC1R risk allele shown to correlate with PTSD severity in females (rs2267735) was found in this study to be associated with worsened anxiety and insomnia symptom severity compared to other females without the risk allele (Ross et al., 2020). In males, levels of PACAP38 and those with the SNP had more mild symptoms than males of other genotypes. This suggests that the PACAP system has sex-specific mechanisms under anxiety-related disorders, which could be mediated in part through the estrogen response element contained within the PAC1R (Ross et al., 2020). These sex-specific differences may contribute to the increased prevalence of anxiety-related disorders in females and potentially also differences in substance use driven by high-anxiety states. However, further research is needed that focuses on the sex-specific factors of PACAP and its impact on anxiety and implications for SUD.

#### Depression

Prepubescent girls and boys show similar rates of depression. However, as puberty begins, the prevalence of depression increases in girls compared to boys. The sex-specific disparity continues into adulthood, with females showing double the rate of depression compared to males (Altemus et al., 2014). Additionally, symptom presentation differs between the sexes, with females showing more comorbid anxiety, eating disorders, gastrointestinal, appetite, weight, and relationship-related symptoms (Altemus et al., 2014). Males with depression show more comorbid SUD and lethal suicide attempts (Altemus et al., 2014). PACAP may underlie the sex differences seen in onset and depression symptomology (Lutfy and Shankar, 2019). For example, an SNP3 (rs1893154) in the *ADCYAP1* gene is associated with major depressive disorder (MDD) in humans, albeit in a non-sex-specific manner (Hashimoto et al., 2010). Alterations in PACAP signaling are linked to changes in rodent behaviors analogous to depression in humans. Similar to stress models and selective optogenetic activation of the BNST, exogenous ICV CNS administration of PACAP in the CNS induces a depressive-like phenotype in male mice, as observed in ICSS, FST, decreased sucrose preference, and reduced social interaction (Seiglie et al., 2015; Hu et al., 2020; Farkas et al., 2017). Similarly, PACAP−/− knockout models show anti-depressive-like behavior in social interaction tests, and social defeat stress-exposed PACAP−/− mice show less immobility time in FST vs. defeated wild-type mice (Hashimoto et al., 2001; Lehmann et al., 2013; Hattori et al., 2012). However, one study found increased immobility and time spent swimming in FST in PACAP−/− mice (Hashimoto et al., 2009). Depressive-like behaviors in sucrose preference and reduced weight gain are associated with changes in neuronal activity and increased PACAP+ cells in the oval nucleus of the BNST in male mice (Hu et al., 2020). In male Wistar rats, ICV administration of PACAP raises the threshold for intracranial self-stimulation (ICSS) (i.e., a decrease in the rewarding properties of the electrical stimulations), thereby implicating an anhedonia phenotype (Seiglie et al., 2015). Anhedonia was then blocked by PACAP(6–38), implanting a role for the peptide and receptor (Seiglie et al., 2015). These studies highlight the importance of PACAP signaling in modulating behavioral changes relevant to the depressive-like phenotype in male rodent models for depression. While PACAP may have a sex-specific role in depression, data on its effects in females are limited. Elevated PACAP immunohistochemical staining was found in the central BNST in post-mortem samples from male subjects with comorbid MDD and bipolar disorder. The elevated PACAP levels were not seen in females (Slabe et al., 2023). This implies PACAP may not play a consistent role across all neuropsychiatric disorders within a given sex, as females with PTSD show elevated PACAP but not in depression (Ressler et al., 2011). Thus, pathological changes in PACAP for males are more associated with depression and stress, while for females, they are associated with anxiety and PTSD (Foilb et al., 2024). As there is a strong link between SUD and depression, the PACAP-driven changes seen in these depression studies could increase the likelihood of SUD in males specifically (Gargiulo et al., 2020).

#### Schizophrenia

Schizophrenia incidence is higher among males and onset is generally earlier in males (Li et al., 2022). Symptom presentation also varies between genders, with males exhibiting cognitive dysfunction, avoidant behaviors, and more pronounced negative symptoms more frequently (Li et al., 2022). Females show more affect-related symptoms, including depression, impulsivity, and emotional instability (Li et al., 2022). Some sex differences in life event-associated onset have been identified, as childhood abuse may be more strongly associated with risk in females vs. males (Li et al., 2022; Garcia et al., 2016). The genetic variant SNP3 of the PACAP gene was found to be associated with schizophrenia, along with reduced hippocampal volume and worsened memory. However, no sex differences were reported, although the majority of subjects studied were male (Hashimoto et al., 2007). A study of postmortem samples found sex and regional differences in PACAP peptide and PAC1R expression in 35 subjects (26 males, 9 females) with schizophrenia who died of either suicide or natural causes (Slabe et al., 2023). In the dorsolateral PFC, PACAP and PAC1R transcripts were significantly upregulated in males with schizophrenia with a natural cause of death and not females with schizophrenia with a natural cause of death. However, when the cause of death was suicide for both sexes, there was no sex difference in PACAP-related genes in the dorsal lateral PFC (Slabe et al., 2023).

It is possible that PACAP plays different roles in males vs. females in schizophrenia, with evidence exhibiting a more active involvement in certain schizophrenia symptomologies in males than in females. Although there are limited studies specifically exploring sex differences in the relationship between schizophrenia and PACAP, the available evidence supports the likelihood of such differences. This notion is particularly compelling given the underrepresentation of females in these studies. The prevalence of SUD is nearly 50% in those with schizophrenia, and PACAP may play a mediatory role, particularly in males (Khokhar et al., 2018).

### PACAP and substance use disorders

**Figure 1:**
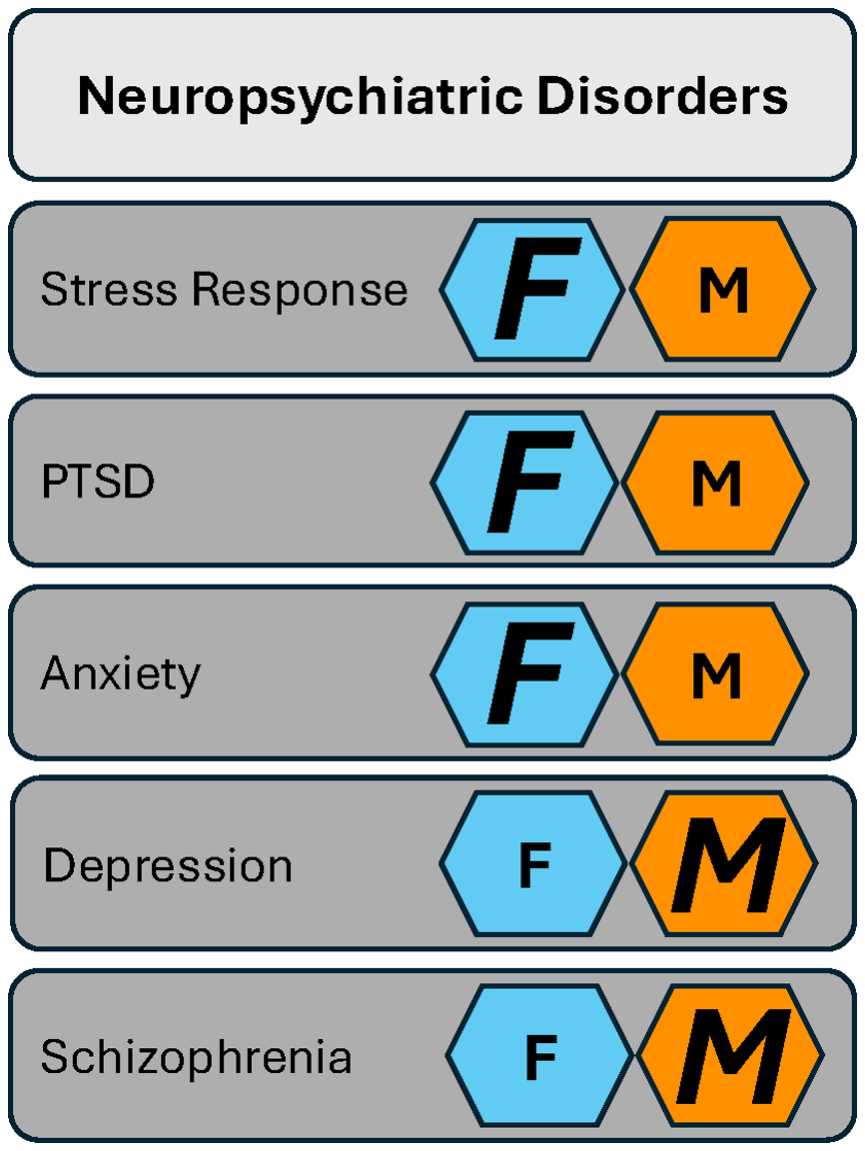
The PACAP system has a role in neuropsychiatric disorders in both females and males. Females (F, dark blue hexagons) are more strongly affected in their biological response to stress and in stress-associated disorders such as PTSD and anxiety. Males (M, orange hexagon) show more pronounced PACAP-related changes in depression and schizophrenia. *Key:* Smaller letters represent evidence in the literature that shows a role for PACAP in a given neuropsychiatric disorder with no bias toward either sex. Larger italicized letters represent the sex more strongly affected.

**Figure 2:**
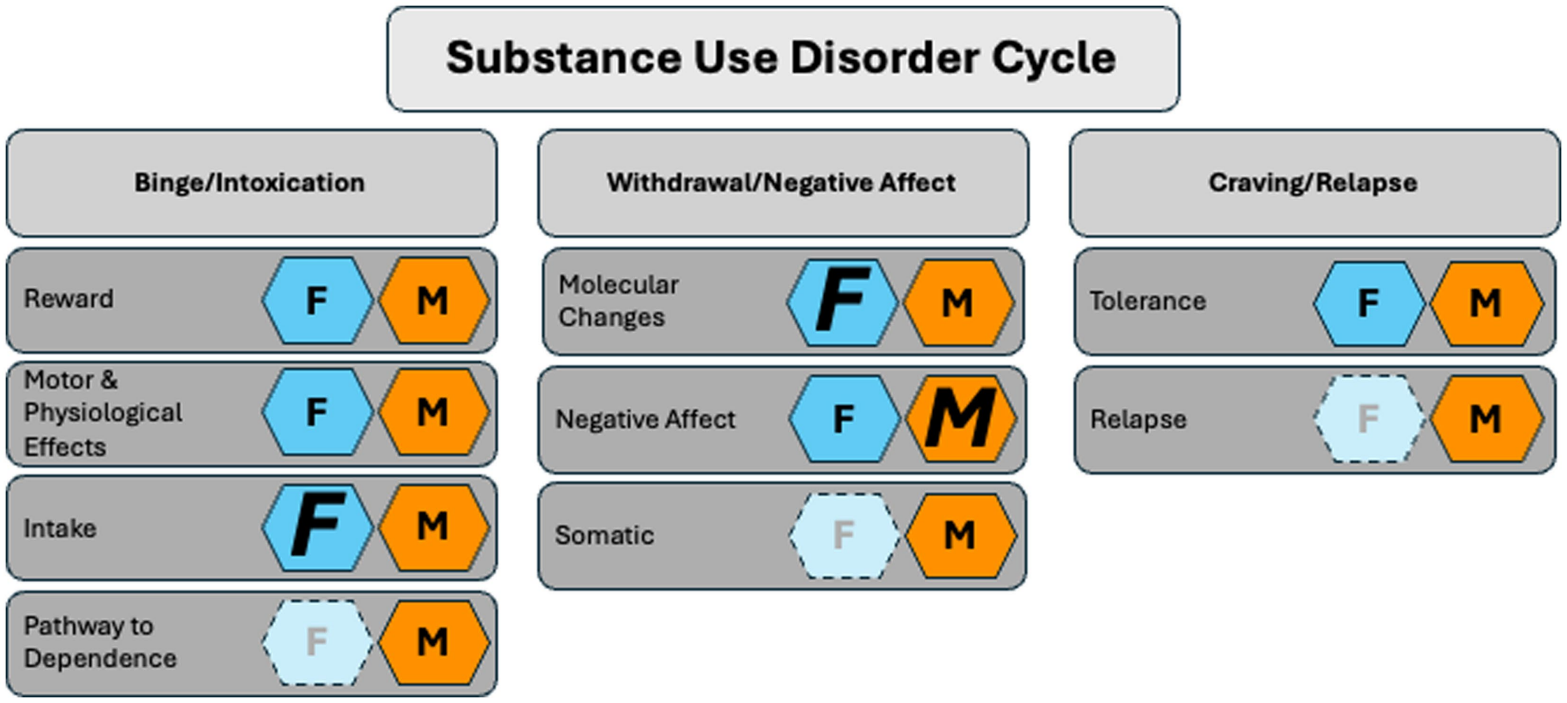
The role of PACAP in the substance use disorder cycle in females and males. Females (F, dark blue hexagon) show heightened sensitivity to PACAP-associated changes in drug intake, withdrawal, and tolerance. Males (M, orange hexagon) exhibit more PACAP-related sensitivity in negative affect. Conclusions are based on current, accessible literature and limited studies (light blue hexagons) in females. Not pictured: the role of PACAP *in vitro* and fetal alcohol syndrome. *Key:* Smaller letters represent evidence in the literature that shows a role for PACAP in a given stage of the SUD cycle with no bias toward either sex. Larger italicized letters represent the sex more strongly affected. The lighter background color and greyed-out letter indicate the absence of literature.

Collectively, these studies on PACAP signaling and dysregulation permeate various neuropsychiatric disorders with sex specificity (see Figure 1). Neuropsychiatric disorders are comorbid with and confer additional risk to the development and maintenance of SUD (Miles et al., 2019; Jones et al., 2023; Pirino and Barson, 2021; Hanson et al., 2011; Substance Abuse and Mental Health Services Administration, 2020). Therefore, understanding sex differences in the cycle of SUD allows for a greater understanding of sex-specific mechanisms that, in turn, can inform tailored therapeutic targets for SUD, including those suffering from comorbid neuropsychiatric disorders. PACAP’s involvement in SUD includes crosstalk between anti-reward processing, stress modulation, and affect-related behaviors (Gargiulo et al., 2020; Stojakovic et al., 2020; Pirino and Barson, 2021). Additionally, PACAP also plays a role in the pathway to drug dependence. Therefore, the literature suggests PACAP is involved in all three stages of addiction: intoxication, withdrawal, and preoccupation/anticipation (Koob and Volkow, 2010). These stages intensify as the use of a particular drug occurs chronically and eventually leads to SUD (Koob and Volkow, 2010). SUD onset occurs during late adolescence in both sexes. Although it is traditionally reported that SUD prevalence is higher in males, the gap has been narrowing (McHugh et al., 2018). There are sex disparities in the severity of SUDs, particularly in the rapid progression of SUD experienced by a subset of females called ‘telescoping.’ Telescoping of SUD in females is seen with alcohol, marijuana, cocaine, and prescription opioids but not heroin (McHugh et al., 2018). Interestingly, sex disparities coincide with sex-specific alterations in PACAP signaling. Here, we attribute characteristics in the PACAP system as it relates to sex differences in the different aspects of the cycle of addiction (see Figure 2).

### Intoxication

#### Reward

Initial drug consumption begins with rewarding effects. Conditioned place preference (CPP) paradigms help demonstrate the rewarding aspect of drugs of abuse by measuring preference for a side associated with the investigator-administered drug. PACAP−/− male and female mice demonstrated blunted morphine CPP with single conditioning but not repeated conditioning relative to wild-type mice (Marquez et al., 2009), suggesting the role of PACAP in the rewarding effects of morphine may be transitory. The PAC1R is not involved in the rewarding effects of morphine, as PAC1R−/− (sex unspecified) mice showed morphine CPP to levels similar to those of their wild counterparts (Martin et al., 2003). Similarly, with nicotine, PACAP−/− females did not change nicotine CPP relative to wild-type mice (Tseng et al., 2019). Recent studies testing females and males confirmed the lack of effect on nicotine CPP (Nega et al., 2020). In ethanol CPP male PACAP−/− showed ethanol preference similar to wild-type mice (Tanaka et al., 2010). The role of PACAP in ethanol CPP remains to be tested in females. These studies suggest PACAP does not have a role in the rewarding effects of drugs of abuse. Interestingly, at higher doses of nicotine, PACAP−/− females demonstrated blunted nicotine CPA (Tseng et al., 2019), demonstrating that the role of PACAP may be specific to drug-induced aversion. Given that ICV PACAP raises the threshold ICSS (Seiglie et al., 2015) suggests that the role of PACAP may be related to anti-reward or aversion. However, more studies are needed to understand the role of PACAP in CPA.

#### Motor and physiological effects

The role of PACAP in the somatic effects of initial exposure to drugs of abuse may be drug-specific. Male PACAP−/− mice relative to wild-type mice demonstrated hyperlocomotion at baseline, but a lack of hyperlocomotion was induced by the investigator- administered amphetamine (Tanaka et al., 2006). The anti-hyperkinetic effect of amphetamine in PACAP−/− mice was dependent on serotonin, as the effect was blocked by a 5-HT1A receptor agonist. The anti-hyperkinetic effect of amphetamine was accompanied by increased neuronal activity in the medial PFC (mPFC) of PACAP−/− mice relative to PACAP controls. There were no differences in amphetamine-induced neuronal activity in the dorsomedial striatum, cingulate cortex, and nucleus accumbens relative to wild-type mice (Tanaka et al., 2006). Altogether, these data suggest amphetamine-induced hyperlocomotion is dependent on PACAP activity that is associated with increased serotonin. Conversely, investigator-administered methamphetamine did not change locomotor activity in male PACAP−/− mice relative to PACAP-intact mice (Fujii et al., 2007). Thus, the lack of effect of methamphetamine compared to amphetamine in males suggests the role of PACAP in stimulant-induced psychomotor activity is sensitive to differences in chemical structure. Interestingly, phenotypes associated with motor deficits may be reversed by stimulants, as at baseline PACAP−/− males demonstrated abnormal jumping behavior that was reduced by amphetamine and methylphenidate (Tanaka et al., 2006), the latter of which is commonly used to treat ADHD. Additionally, at baseline, PACAP−/− mice demonstrates a pre-pulse inhibition deficit, a phenotype associated with schizophrenia, that was reversed to control levels with amphetamine (Tanaka et al., 2006). Altogether, the data suggest a role for PACAP in amphetamine-induced psychomotor effects and implicate a role for the schizophrenic-like phenotype in males.

Unlike amphetamine, investigator-administered morphine did induce hyperlocomotion, albeit at reduced levels in male and female PACAP−/− mice relative to wild-type mice (Marquez et al., 2009), suggesting a modulatory role of PACAP on morphine-induced hyperlocomotion. Indeed, in C57BL/6J male and female mice, administering low-dose PACAP ICV demonstrated potentiation of morphine-induced hyperlocomotion relative to aCSF controls (Marquez et al., 2009), thus suggesting PACAP is sufficient to modulate morphine-induced hyperlocomotion. However, in PAC1R−/− (sex unspecified), acute repeated morphine exposure did not induce hyperlocomotion relative to wild-type mice (Martin et al., 2003). At baseline, both PACAP−/− and PAC1R−/− mice demonstrated increased locomotion relative to wild-type controls. Given that morphine only induces hyperlocomotion in PACAP−/− mice suggests a modulatory role of PACAP while PAC1R may be necessary for morphine-induced psychomotor effects. Alternatively, the difference in dose and administration regimen suggests morphine-induced hyperlocomotion is dose-dependent in mice with a deficient PACAP system.

In regard to drug-induced sensorimotor impairment, ICV administration of PACAP had no effects on EtOH-induced loss of righting reflex in males (Szabó et al., 1998). Similarly, PAC1R−/− males demonstrated no change in EtOH-induced righting reflex (Otto et al., 2001). However, PACAP−/− males demonstrated reduced loss of righting reflex to ethanol (Otto et al., 2001; Tanaka et al., 2004). The lack of effect in PACAP−/− mice and decrease in PAC1R−/− mice suggests PACAP is not necessary, although PAC1R can modulate the loss of the righting reflex induced by ethanol. However, the role of PACAP may be species-and gene-dependent, as Drosophila males with a mutation in *amnesiac*, a gene with weak homology to PACAP, showed enhanced ethanol-induced loss of postural control (Moore et al., 1998). These data suggest that the PACAP system plays a role in the ethanol-induced sensorimotor effect in males. Future studies are needed to understand the role of PACAP on drug-induced sensorimotor effects in females.

The role of PACAP in the effects of drug intoxication extends to ethanol-induced physiological responses, as a single ICV treatment of PACAP enhanced the hypothermic effects of high-dose ethanol in CFLP male mice. PACAP−/− males demonstrated blunted ethanol-induced hypothermia dependent on serotonin, as a 5HT1A antagonist restored hypothermia relative to wildtype levels (Tanaka et al., 2010; Tanaka et al., 2004). Altogether, this suggests PACAP modulates but is not necessary for ethanol-induced hyperthermia and is partly mediated by serotonin. Interestingly, a history of repeated ICV administration of PACAP decreased the hypothermic effect of a high dose of ethanol (Szabó et al., 1998), suggesting that adaptations occur in the PACAP system that result in blunted physiological effects of initial exposure to ethanol. These studies suggest that PACAP is sufficient to modulate ethanol-induced hyperthermia, and a disturbance in baseline PACAP levels can block this effect. Overall, the PACAP system plays a role in the psychomotor effects of amphetamine and morphine and the sensorimotor and physiological effects of ethanol. While further studies on females are needed, current findings indicate no significant differences between females and males.

#### Intake

Clinical data in non-dependent individuals demonstrate polymorphism in *ADCYAP1* of socially drinking males and females associated with alcohol consumption (Kovanen et al., 2010). In females, the differences in PACAP polymorphisms also extend to problematic drinking and in subjects with cluster headaches. Alcohol consumption triggers headaches more frequently in males, which may passively drive drinking in females specifically (Mahović and Bračić, 2023; Dragan et al., 2017). Preclinical studies reviewed in (Stojakovic et al., 2020) suggest a role for PACAP in modulating intake using home-cage access and self-administration models. Studies in females using a two-bottle choice (2BC) method, a model of continuous free access to drug and water, demonstrate PACAP−/− mice consumed more nicotine and had a dose-dependent higher preference for nicotine relative to wild-type mice (Tseng et al., 2019). The effect of endogenous PACAP on intake extends to ethanol, as PACAP−/− males demonstrated increased preference and intake for ethanol over water in 2BC relative to wild-type mice (Tanaka et al., 2010). Additionally, the increased intake was selective for ethanol, as PACAP−/− males did not demonstrate changes in sucrose and quinine intake relative to their wild-type counterparts (Tanaka et al., 2010). To the best of our knowledge, nicotine intake has not yet been measured in PACAP−/− males, and alcohol intake has not been assessed in females using the 2BC method. Taken together, these studies demonstrate that the absence of endogenous PACAP drives a preference for nicotine in females and ethanol drinking in males.

Studies using intermittent two-bottle choice drinking (IA2BC) reveal sex differences, particularly highlighting greater ethanol consumption and preference in females. Notably, despite higher drinking levels in females in IA2BC, chemogenetically inhibiting PACAP projections to the BNST reduces ethanol consumption in both sexes (Lepeak et al., 2023), suggesting a sex-independent sensitivity to PACAP perturbations. Furthermore, Gargiulo et al., 2021 demonstrated that modulating the rostral nucleus accumbens shell (NAsh) with PACAP27 but not PACAP38 reduces early-onset intake in both males and females, with a more prolonged effect observed in females. These studies demonstrate the influence of PACAP on drinking behavior is isoform-specific and that females exhibit heightened sensitivity to such modulation. The specificity of this effect was further supported by antagonists, as only PACAP(6–27), and not PACAP(6–38), was found to increase early onset intake in the NAsh without affecting sucrose consumption. Additionally, the isoform-specific role of PACAP is also region-specific. In the nucleus accumbens core (NAcc), PACAP38 decreased drinking, while PACAP27 had no effect (Gargiulo et al., 2021). These studies collectively highlight an isoform-and region-specific role of PACAP in modulating drinking intake, with females showing increased sensitivity across the BNST, NAsh, and NAcc.

#### Pathway to dependence

Mouse lines that are bred to model higher alcohol intake also show baseline differences in PACAP expression. Alcohol-preferring male rats display higher levels of PAC1R receptor in the NAcc relative to wild-type mice (Minnig et al., 2022). The role of PAC1R is confirmed functionally, as ICV and intra-NAcc administration of the PACAP(6–38) receptor antagonist decreased motivation and seeking behavior in alcohol-preferring male rats relative to the non-alcohol-preferring (Minnig et al., 2022). Moreover, the decrease in self-administration was specific to ethanol, as sucrose or saccharine self-administration did not differ with PACAP(6–38) receptor antagonist (Minnig et al., 2022). shRNA knockdown of PAC1R in the NAcc prevents increased levels of ethanol intake in alcohol-preferring male rats (Minnig et al., 2022). The effects of PAC1R knockdown are region-specific, as in the NAsh, knockdown increases motivation and ethanol self-administration in alcohol-preferring male rats (Minnig et al., 2021). The role of PAC1R in alcohol-preferring females remains to be tested. The chronic intermittent ethanol vapor exposure (CIE) model increases ethanol self-administration and provides insight into dependence. In the CIE model, the PAC1R antagonist in the BNST in males inhibits the CIE-induced increase in self-administration (Ferragud et al., 2021). The role of PACAP in heightened drug intake extends to cocaine self-administration. PACAP transcript levels in BNST increase with cocaine self-administration in male high-responders and not with an acute cocaine intake (Miles et al., 2018). Altogether, data suggest that increased PAC1R and PACAP in the NAcc and BNST, respectively, are associated with an increased drinking phenotype. Additionally, it demonstrates a functional role for PAC1R in the NAcc, NAsh, and BNST in modulating changes in heightened drinking behavior.

### Withdrawal and abstinence

#### Molecular changes

Repeated withdrawal cycles are a hallmark of addiction. Recent studies show that early withdrawal from IA2BC increases PACAP levels in the BNST in both males and females, with a higher increase observed in females (Lepeak et al., 2023). However, PAC1R levels remain unaffected during this early withdrawal phase (Lepeak et al., 2023), suggesting that withdrawal-induced changes are specific to the peptide itself. In males, similar findings are reported during acute withdrawal from CIE exposure, where PACAP levels rise in the BNST without any corresponding change in PAC1R levels. Notably, no changes in PACAP or PAC1R expression were observed in the CeA in males during CIE withdrawal (Ferragud et al., 2021), suggesting that the PACAP system’s response to withdrawal is specific to the BNST and not the CeA. Furthermore, the changes in PACAP levels in the BNST may be transient during withdrawal or vary depending on the drug, as evidenced by the stable PACAP levels during prolonged abstinence from cocaine self-administration in males (Miles et al., 2018). Although PACAP levels remain unchanged in cocaine abstinence, there is evidence to suggest that PACAP may still play a role during cue-induced reinstatement. It is unknown if the lack of changes in PACAP expression in the BNST are also not present in females during abstinence from cocaine self-administration.

Studies using withdrawal from investigator-administered drugs demonstrate a lack of effects associated with PACAP in withdrawal in other brain regions. During acute withdrawal from morphine, PAC1R−/− mice (sex unspecified) demonstrated increased firing rates in the locus coeruleus at similar levels as wild-type mice (Martin et al., 2003), suggesting these neuroadaptations are not specific to the PACAP system in the locus coeruleus. In PACAP−/− males, prolonged withdrawal from methamphetamine does not change basal levels of extracellular dopamine and serotonin levels in the mPFC in both PACAP−/− and wild-type mice (Fujii et al., 2007), suggesting mPFC is unaffected in methamphetamine withdrawal. However, we note below that neuroadaptations associated with PACAP in the mPFC are revealed with re-exposure to methamphetamine post-abstinence. Other studies investigating withdrawal from experimenter-administered cocaine in males demonstrate increased PACAP and PAC1R mRNA in the hippocampus, an effect that is still present 14 days post-withdrawal (Mai et al., 2018). Interestingly, the cocaine-induced increase in PACAP and PAC1R is blunted in interleukin-6-deficient (IL-6) KO mice, suggesting the cocaine-induced increase is mediated through the immune system. The role of PACAP in various other brain regions and drugs of abuse remains to be tested, particularly in females. Altogether the current literature demonstrates changes in the PACAP system within the BNST during withdrawal from ethanol and hippocampus in withdrawal from cocaine. Additionally, the role of PACAP in the BNST has sex-specific implications, with females demonstrating increased sensitivity to PACAP perturbations.

#### Negative affect

Following chronic drug administration, patterns of consumption change that are often associated with increased negative affect. As discussed above, infusions of PACAP directly into the BNST increase anxiety-like behaviors, and PACAP−/− mice show anxiolytic responses in males (Lezak et al., 2014; Hammack et al., 2009; Hattori et al., 2012; Martelle et al., 2021). Given the relationship between PACAP and anxiety-like and aversive states, it follows that the negative affect associated with SUD would be mediated by PACAP in a sex-specific manner. To date, the role of PACAP on anxiety-like behavior has been shown in withdrawal from ethanol, nicotine, and cocaine. Specifically, a recent study found that males and not female PACAP−/− mice demonstrated increased anxiety-like behavior in the elevated plus maze in mecamylamine-induced nicotine withdrawal (Nega et al., 2020), suggesting males may be more sensitive to perturbations in the endogenous PACAP system as they relate to anxiety during nicotine withdrawal.

However, in males, the exogenous ICV administration of PACAP did not change EPM behavior relative to aCSF in naloxone-induced morphine withdrawal (Lipták et al., 2012), suggesting that PACAP does not have an anxiolytic effect in the context of morphine exposure. The role of PACAP on negative affect in ethanol withdrawal is specific to a dependent state, as intra-BNST administration of PACAP(6–38) decreased anxiogenic behavior in male rats exposed to CIE and not air vapor controls (Ferragud et al., 2021). The effect of PACAP on withdrawal-induced anxiety in dependent vs. non-dependent females remains to be tested. Interestingly, co-administration of PACAP with ethanol has developmental effects in a model of fetal alcohol syndrome. Intrauterine co-administration of PACAP with ethanol blunted the ethanol-induced anxiety-like behavior in the EPM at P30 (Shili et al., 2021), suggesting a protective role of PACAP in utero. Altogether, the studies suggest a role for PACAP in modulating anxiety-like behavior in males exposed to ethanol and nicotine.

#### Somatic

Historically, papers have investigated the role of PACAP on withdrawal in the context of morphine. Further investigation of the role of PACAP on the somatic effects induced by morphine withdrawal is needed, as the current literature suggests different roles for PACAP that may be drug-dose-specific or dependent on the mouse strains (Stojakovic et al., 2020). Using naloxone in prolonged abstinence to precipitate morphine withdrawal demonstrates PACAP−/− mice (sex unspecified) have a heightened withdrawal score as measured with loss of body weight, jumps, wet dog shakes, paw tremor, sniffing, body tremor, ptosis, diarrhea, teeth chattering, and piloerection relative to wild-type mice (Martin et al., 2003). The anti-withdrawal role of PACAP was confirmed functionally and with exogenous administration in wild-type mice (sex unspecified), as a single ICV administration of PACAP-38 prior to naloxone administration decreased physical withdrawal score (Martin et al., 2003). Conversely, measuring naloxone-precipitated withdrawal with withdrawal jumping and hypothermia in CFLP mice (C57BL/6-FVB hybrid strain) demonstrates that PACAP-38 had a pro-withdrawal effect in males (Lipták et al., 2012). Similarly, using the CFLP mice strain, albeit with repeated co-administration of PACAP-38 ICV, demonstrated a pro-withdrawal effect, as it facilitated naloxone-precipitated withdrawal jumping in males (Mácsai et al., 2002), suggesting the role of PACAP in withdrawal may be specific to mouse strains. However, in another study also using CFLP mice and withdrawal jumping to measure naloxone-precipitated withdrawal, repeated administration, albeit with a lower dose of ICV administration of PACAP, demonstrates PACAP had no effects on withdrawal (Szabó et al., 1998), suggesting the role of PACAP may also be dose-dependent. Altogether, the studies suggest a complex role of PACAP on morphine withdrawal in males. Studies using females are needed to further expand our understanding of PACAP on drug-induced somatic effects.

### Tolerance

Repeated exposure to drugs can induce tolerance that is manifested as an adaptation to drug-associated somatic effects. The changes that occur in the PACAP system on the pathway to dependence and its role in the initial somatic responses to initial drug intake suggest that PACAP may also modulate somatic symptoms of tolerance. Daily ICV administration of PACAP38 in a model of chronic morphine exposure facilitated tolerance measured via an antinociceptive response in males (Mácsai et al., 2002). Conversely, ICV administration of PACAP did not affect the development of tolerance in males, as measured by analgesia (Szabó et al., 1998), suggesting that the effect of PACAP on tolerance is dose-dependent. A study investigating the effects of PACAP after developing tolerance (i.e., post-tolerance and not co-administered with morphine) demonstrates that IP administration of the PACAP antagonist, M65, blocked opioid-exaggerated cortical spreading depression, which models migraine aura, in female migraine models (Bertels et al., 2023). This finding suggests a significant role for the PAC1R in females. Furthermore, using hyperlocomotion as a measure of morphine sensitization demonstrates a phenotype-specific effect of PACAP. As mentioned, a lack of endogenous PACAP results in heightened locomotion in PAC1R−/− mice. Notably, despite this hyperlocomotion, PAC1R−/− mice (sex unspecified) demonstrated regular sensitization to the locomotor effects of repeated morphine administration compared to wild-type mice (Martin et al., 2003). Similarly, in a model of methamphetamine tolerance, PACAP−/− males showed similar behavioral sensitization comparable to that of PACAP-intact controls (Fujii et al., 2007). Given that PACAP also had no psychomotor effect upon initial methamphetamine exposure (Fujii et al., 2007), this suggests that PACAP does not modulate the psychomotor effects in the cycle of addiction. Altogether, these studies suggest a role for the PACAP system in the nociceptive and not psychomotor effects of opioid tolerance. Future studies must investigate if PACAP does not affect tolerance-induced hyperlocomotion in females.

Repeated exposure to drugs of abuse can increase the probability of seizures (i.e., the kindling effect). Data suggest that the PACAP system may be protective against cocaine-induced kindling in males. Studies demonstrate daily ICV administration of PACAP(6–38) facilitated cocaine-induced convulsions in male mice (Mai et al., 2018). The kindling effect of PACAP(6–38) occurred in the presence of the anti-convulsive IL-6, suggesting blocking the PACAP system activity induces kindling. This is further supported in IL-6 KO mice, as they demonstrate increased sensitivity to cocaine-induced convulsions that are accompanied by increased PACAP and PAC1R mRNA levels in the hippocampus (Mai et al., 2018). Thus, activating PACAP may be protective against kindling, as the administration of IL-6 upregulates PACAP, partly through activation of JAK2/STAT3 (Mai et al., 2018). Moreover, IL-6 blocked the cocaine-induced decrease of anti-apoptotic proteins, Bcl2 and Ccl-XL, which was reversed by PACAP(6–38) (Mai et al., 2018), suggesting the protective effects of IL-6 are mediated via endogenous PACAP (Mai et al., 2018). These data demonstrate that activating the PACAP system is neuroprotective of cocaine-induced kindling in males. Future studies are needed to investigate the neuroprotective role of PACAP in females.

### Relapse

Abstinence from drug intake can heighten anticipation and preoccupation with drugs of abuse, driving an individual to relapse. Previous studies reviewed in Gargiulo et al. (2020) and Miles et al. (2019) suggest inhibiting PACAP can block reinstatement of drug-seeking behavior. Specifically, Miles et al. (2018) demonstrated that after extinction training, administration of PACAP(6–38) antagonist in the BNST blunted stress-induced reinstatement in males. Conversely, PACAP38 peptide infusion in the BNST induced cocaine seeking in male rats (Miles et al., 2018), suggesting that PACAP has a similar effect as a stressor in drug reinstatement. In a model of investigator-administered methamphetamine, re-exposure after prolonged withdrawal increased extracellular serotonin and not dopamine levels in the mPFC in both PACAP−/− and wild-type males (Fujii et al., 2007). These studies suggest the role of PACAP in relapse-like phenotypes may be limited to the BNST or stimulant-specific. Future studies will need to investigate the role of PACAP in other brain regions during the reinstatement of various drugs of abuse and the effect on females.

### Fetal alcohol syndrome

Exposure to alcohol in utero and postnatally can have deleterious effects. As mentioned above, PACAP plays a role in development in utero and after birth and thus may be dysregulated with drug exposure during critical periods of development. Interestingly, the role of PACAP during development may be protective against the deleterious effects of drug exposure. Daily intrauterine administration of a moderate dose of ethanol (Shili et al., 2021) from gestation to parturition induces alcohol-induced oxidative stress, neuronal cell death, motor activity, and anxiety-like behavior at postnatal day 30 (Shili et al., 2021). However, intrauterine co-administration of PACAP with ethanol in utero blunts the toxic effects at P30 (sex unspecified) (Shili et al., 2021). PACAP was administered postnatally in the subarachnoid space above the cerebellum of ethanol-exposed PND8 age rats (sex unspecified), approximately the age of a human infant (Semple et al., 2013), increased markers of neuronal differentiation and increased cells in the cerebellum cortical layer. Moreover, co-administration of PACAP blocked the ethanol-induced transient increase in pro-apoptotic mRNA and decreased cerebellum cells and cortical layer thickness (Botia et al., 2011). The latter effect on cortical thickness was mimicked by c-jun N-terminal kinase (JNK) and caspase three, suggesting they may underlie the protective effects of PACAP. Additionally, pretreatment with PACAP was protective against the negative geotaxis effect induced by ethanol (Botia et al., 2011). These neuroprotective effects of PACAP are also seen in adolescence, as PACAP−/− mice (sex unspecified) exposed to one binge-like episode at P30 or P95 experienced more oxidative stress and cell death with age-specific changes in ethanol-induced gene dysregulation (Lacaille et al., 2017). Altogether, the studies suggest a neuroprotective role for PACAP in fetal alcohol syndrome. Future studies will need to investigate the role of PACAP on other drugs of abuse in utero and adolescence. Additionally, the sex-specific differences in the role of PACAP on neuroendocrinology suggest future studies are needed to investigate if PACAP sex-specifically contributes to the neuroprotective effects.

### *In vitro* exposure

Similar to in utero and adolescence, PACAP has been shown to have neuroprotective effects on ethanol-induced abnormalities *in vitro* (Reglodi et al., 2019). In cerebellar granule cells, co-and post-exposure to PACAP can reverse alcohol-induced oxidative stress and promote cell survival (Vaudry et al., 2002). The neuroprotective contribution of endogenous PACAP has been shown as granule neurons from PACAP−/− mice demonstrated increased sensitivity to ethanol-induced toxicity (Vaudry et al., 2002). Moreover, Manavalan et al., 2017 demonstrated that PACAP-blunted ethanol-induced cytotoxicity occurs through PAC1R in neuroblastoma cells. Acute exposure to ethanol increases PAC1R mRNA levels via the nuclear compartmentalization of the scaffolding protein Receptor for Activating C Kinase 1 (RACK1) (He et al., 2002). At the neurophysiological level, PACAP blocked the ethanol-induced decreases in resting membrane potential at the neurophysiological level by inhibiting potassium rectifying currents through PAC1R (Castel et al., 2006; Mei et al., 2004). Altogether, this demonstrates the neuroprotective role of endogenous PACAP, which is mediated by PAC1R. The role of PACAP also extends to co-drug exposure, as PACAP blunts cell death induced by the co-administration of ethanol and nicotine in neuroblastoma cells (Manavalan et al., 2017). Given that *in vitro* PACAP has neuroprotective effects in cerebellar neurons, it further demonstrates the need to investigate PACAP in SUD.

## Conclusion

Based on the reviewed literature, it is clear that the endogenous PACAP system is affected in addiction contexts. Modulation of the PACAP system demonstrates the system may be a potential target for treating SUD. The role of PACAP is shown in nicotine, ethanol, stimulant, and opioid studies. In general, the contribution of PACAP to SUD is more pronounced in the intoxication and withdrawal cycles of addiction. During initial drug exposure, studies in males demonstrated that PACAP is involved in the psychomotor effects of stimulants and opioids, as well as the physiological effects of ethanol. Ethanol and nicotine intake are also modulated via PACAP, with females demonstrating heightened sensitivity. PACAP is also involved in the tolerance and dependence of morphine, cocaine, and ethanol in males. Studies in males show that PACAP has a role in negative affect and physiological and molecular changes induced by withdrawal from nicotine, ethanol, morphine, and stimulants. Moreover, male studies implicate the role of PACAP in cocaine relapse. Altogether, these studies support the role of PACAP across a variety of drugs of abuse. Of note, not all drugs and SUD cycles involve the PACAP system (e.g., methamphetamine relapse and tolerance, nicotine and ethanol reward), thereby emphasizing its complex role.

PAC1R expression and PACAP mRNA and peptide are widely present throughout the brain, with particular emphasis on the BNST, amygdala, hypothalamus, and thalamus (Hirabayashi et al., 2018; Hammack et al., 2010; Arimura et al., 1991; Martelle et al., 2021; Hannibal, 2002). Interestingly, the role of PACAP in SUD appears to be brain region-selective. Despite abundant PACAP+ projections to the CeA and the role of CeA in stress and SUD, studies show no changes in expression in ethanol withdrawal (Lepeak et al., 2023; Ferragud et al., 2021). However, functional studies are needed to confirm that CeA is not associated with PACAP’s role in SUD. Conversely, female sensitivity is seen in the PACAP system of NAsh and NAcc associated with alcohol intake. The PACAP system in the BNST has a role in the SUD cycle, specifically in alcohol intake and withdrawal and cocaine intake and relapse. Moreover, the sex-dimorphic role of BNST also extends to the PACAP system, as females showed increased sensitivity associated with alcohol intake and withdrawal. It is unknown if the sex-dimorphic role of PACAP in the BNST also extends to withdrawal. Future SUD studies are needed to investigate the source of PACAP in the BNST, such as the PVT and PBN, as they demonstrate sex-specific characteristics (Curtis et al., 2023; Jaramillo et al., 2020). In cocaine, the mPFC is involved in tolerance and the hippocampus in withdrawal, showing the widespread nature of PACAP’s role in the SUD cycle. The cerebellum appears to be involved in the role of fetal alcohol syndrome and the neuroprotective aspect of PACAP. The neuroprotective aspect of PACAP is emphasized during development but also post-stroke or traumatic brain injury in adults (Jiang et al., 2023; Farkas et al., 2004), perhaps demonstrating time-and event-specific neuroprotection. Implications of this may translate to new PACAP-centric therapies for SUD, although more research is needed in this area.

The limited studies that include females demonstrate that females are more sensitive to changes induced by the PACAP system in SUD. Specifically, females demonstrate heightened PACAP-induced changes in ethanol drinking and withdrawal and morphine tolerance. Female-only studies display a role for PACAP in nicotine intake. Interestingly, the negative affect in females is not affected by PACAP modulation during nicotine withdrawal, albeit it is in males. The lack of change in negative affect is surprising given the evident sex-specific differences in the role of PACAP on affect in neuropsychiatric disorders. While the literature supports PACAP-related sensitivity to negative effects during withdrawal in males, other stress-related aspects, such as intake, show female sensitivity. Perhaps PACAP in females has a role in negative affect that are specific to intake and contribute to the pathway to dependence. For example, females tend to drink to alleviate negative affect, specifically to lower stress and/or depressive states, whereas males drink more for a euphoric experience (Becker et al., 2012). Thus, a proposed role of PACAP in females would be to promote negative affect and contribute to negative reinforcement in both initial intake and relapse (Miles et al., 2019; Pirino and Barson, 2021). Measuring intake and withdrawal-induced molecular changes in IA2BC collectively showed females have increased sensitivity to PACAP system disturbances in the BNST, which is highly related to stress disorders in females (Ressler et al., 2011; King et al., 2017; Kiss et al., 2007). Stress is a known contributor to SUD, and while SUD is more common in males, females develop substance misuse behaviors faster than males do (Bangasser and Valentino, 2014).

Additionally, there is a significant lack of studies in females assessing negative affect across multiple drugs of abuse, as only one study assessed sex differences in aspects of withdrawal-related negative affect. There could also be sex-and drug-specific responses, as male PACAP−/− mice showed reduced anxiety-like behaviors with nicotine withdrawal, but males given ICV PACAP during morphine withdrawal did not (Nega et al., 2020; Lipták et al., 2012). Drug-specific responses indicate that females could react differentially as well; however, this is unknown. Responses to aversive stimuli tie together emotional regulation, stress-related disorders, and the dysregulated aversive emotional processing that occurs in SUD (Muench et al., 2021). PACAP has a prominent role in processing aversive stimuli, including fear-and avoidance-related stimuli, as well as its established roles in stress and anxiety that are closely tied to substance abuse risk (Boucher et al., 2021; Gargiulo et al., 2020; Cleck and Blendy, 2008). For example, PACAP+ neurons are activated by aversive stimuli, and activation of those neurons elicits a panic-like phenotype in mice of both sexes (Kang et al., 2024). Thus, the role of PACAP in avoidance and aversion could also contribute to the pathway to dependence and SUD (Rajbhandari et al., 2023; Boucher et al., 2021; Gilmartin and Ferrara, 2021). Certainly, more studies in females are needed to fully understand the higher sensitivity to PACAP changes in stress-related disorders vs. SUD.

The stress response pathways activated after chronic drug exposure reduce reward system activation, thereby recruiting the ‘anti-reward system’ (Miles et al., 2019; Koob and Le Moal, 2008). The ‘anti-reward system’ is hypothesized as being a series of neuroplastic processes that limit reward and contribute to an aversive and stressful state (Koob and Le Moal, 2008). The literature reviewed suggests PACAP is likely involved in the anti-reward system in SUD (Miles et al., 2019). The lack of effect on CPP induced by PACAP disturbances suggests that PACAP does not play a role in reward, at least in males (Gargiulo et al., 2020; Tanaka et al., 2010).

Given that PACAP disturbances in females have blunt nicotine effects, CPA emphasizes PACAP’s role as an ‘anti-reward system’ in females (Miles et al., 2019). In a drug-naïve state, chemogenetic activation of PACAP+ neurons in the lateral habenula induced CPP in both sexes (Levinstein et al., 2022), while ICV PACAP increases the threshold for ICSS in male rats (Seiglie et al., 2015). The brain regions modulating the role of drug-CPP remain to be investigated. However, a similar brain-region-specific effect may occur regarding the rewarding effects of drugs of abuse given PACAP in the NAcc and NAsh modulate ethanol intake (with female sensitivity) and dependence with opposite effects. Perhaps the presence of a drug and/or drug-dependent state is vital in eliciting anti-reward and is region-specific.

Care was taken to ensure that the literature was adequately balanced and representative of the field in a non-biased manner. For example, when applicable, all studies, regardless of contradictory results, were presented (e.g., section on depression and tolerance). However, there are a few important considerations and limitations in the context of this review. One such consideration is regarding knockout models, as PACAP−/− genetic knockouts in C57Bl6/N mice can result in consequential downstream gene expression changes (Bakalar et al., 2021). Thus, the recruitment or downregulation of nearby genes may explain some of the conflicting evidence derived from exogenous manipulation of the PACAP system relative to the genetic knockdown of PACAP or PAC1R. Brain region-specific administration vs. ICV PACAP may also explain differences in results between studies, as the latter induces a global CNS effect. Therefore, ICV administration may recruit additional brain regions that were not considered in the present review. An additional consideration is the limited studies that tested both PACAP27 and PACAP38. Given PACAP isoforms have differential expression and brain region-specific effects, studying PACAP27 and PACAP38 simultaneously will provide a more comprehensive understanding of the role of the PACAP system (Vaudry et al., 2009; Arimura et al., 1991; Amin and Schytz, 2018). A limitation in our interpretation is the focus on PAC1R. PACAP can bind to VPAC1R and VPAC2R in addition to PAC1R. Therefore, in studies that did not isolate PAC1R, the role of the PACAP should be considered in the context of all three receptors. Another point to consider is the limited number of studies on females compared to males. While the focus of this review was on stress, PTSD, anxiety, depression, and schizophrenia, the sex-dimorphic role of PACAP may extend to other disorders (e.g., eating disorders and multiple sclerosis). Thus, studies investigating other diseases comparing males vs. females can provide a more informed role of the sex-dimorphic role of the PACAP system. Additionally, the present review does not cover all drugs of abuse, as it was limited to the available studies investigating nicotine, ethanol, amphetamines, cocaine, and opioids. Further investigation into additional drugs (e.g., cannabis) would be beneficial as they may provide additional support or unveil the role of PACAP in other phases of the SUD cycle.

To conclude, the evidence reviewed above indicates there is a complicated relationship between stress, PACAP signaling, and sex differences in the response to stress-related disorders and SUD. Stress can increase the likelihood of initial drug use and increase drug-seeking behaviors, cravings, and the risk of relapse. Thus, stress acts in multiple ways to increase the propensity toward drug use and/or perpetuate the cycle. Activation of the HPA axis is seen with acute drug administration in a drug-specific manner; generally increases with alcohol, nicotine, and cocaine and decreases with opioids. Changes in the HPA axis are modified over time, similarly to how chronic stress affects the HPA axis (Cleck and Blendy, 2008). It is hypothesized that PACAP signaling with repeated insult increases stress, anxiety, and negative affect and drives increased drug use and/or relapse from a negative reinforcement lens. Perturbations in PACAP could be exaggerated in females and/or uniquely modifiable for females with cycling sex hormones (Gargiulo et al., 2020). The studies in PTSD demonstrated a role for estrogen to modify the PAC1R gene, and this could have effects on SUD risk and progression. Studying PACAP in the context of neuropsychiatric disorders and SUD has revealed sex dimorphisms, broadening our understanding and opening new avenues for precision treatment of disorders that are not experienced equally by the sexes.
